# A New Model for Predicting Rate of Penetration Using an Artificial Neural Network

**DOI:** 10.3390/s20072058

**Published:** 2020-04-06

**Authors:** Salaheldin Elkatatny, Ahmed Al-AbdulJabbar, Khaled Abdelgawad

**Affiliations:** College of Petroleum Engineering & Geosciences, King Fahd University of Petroleum & Minerals, Dhahran 31261, Saudi Arabia; g200679600@kfupm.edu.sa (A.A.-A.); abouzidan@kfupm.edu.sa (K.A.)

**Keywords:** artificial neural networks, rate of penetration, drilling parameters, ROP empirical correlation

## Abstract

The drilling rate of penetration (ROP) is defined as the speed of drilling through rock under the bit. ROP is affected by different interconnected factors, which makes it very difficult to infer the mutual effect of each individual parameter. A robust ROP is required to understand the complexity of the drilling process. Therefore, an artificial neural network (ANN) is used to predict ROP and capture the effect of the changes in the drilling parameters. Field data (4525 points) from three vertical onshore wells drilled in the same formation using the same conventional bottom hole assembly were used to train, test, and validate the ANN model. Data from Well A (1528 points) were utilized to train and test the model with a 70/30 data ratio. Data from Well B and Well C were used to test the model. An empirical equation was derived based on the weights and biases of the optimized ANN model and compared with four ROP models using the data set of Well C. The developed ANN model accurately predicted the ROP with a correlation coefficient (R) of 0.94 and an average absolute percentage error (AAPE) of 8.6%. The developed ANN model outperformed four existing models with the lowest AAPE and highest R value.

## 1. Introduction

The drilling rate of penetration (ROP) is a measure of the speed or the progress of the drill bit when it drills subsurface formation. ROP is usually reported in ft/h (field units) or m/h (SI units). The major portion of the well capital investment is consumed by drilling operations; thus, optimizing the ROP is a key aspect to reduce total well cost [1,2,3]. ROP modeling challenges arise from the fact that ROP is affected by many interconnected factors, which makes it very difficult to infer the mutual effect of each individual parameter. As a result, many oil and gas companies maintain data for the offset wells in the same field and set certain key performance indicators to assess the ROP for any newly drilled well [4].

In order to drill a well, three factors have to be established together. First of all, a certain load has to be applied on the bit, and this is known as the weight on bit (WOB). WOB can be achieved by the rig hoisting system by slacking some weight of the drill string against the hole being drilled. The drillstring rotation speed by the rig rotary equipment, such as the top drive or the kelly in older rigs, is measured in revolutions per minute (RPM). The action of WOB and drillstring rotation generates a torque (T) as a result of the interaction between the drilling bit and the drilled formation in addition to friction with the wellbore wall [5]. Finally, a drilling fluid has to be circulated within the wellbore and using the rig circulating system to clean the wellbore and cool the drill bit at circulation rate (Q). Surface pressure is generated at the stand pipe against any pressure losses during the mud circulation (friction, hydrostatic, drill bit nozzles ΔP, etc.). This pressure is known as stand pipe pressure (SPP) [6]. The combination of many drilling parameters, such as WOB, drillstring rotation, torque, mud circulation rate (Q), bit type, and bit hydraulics, defines the performance of the drilling operation. Usually, the main objective of a successful drilling operation is to optimize these parameters to increase ROP and make the operation cost-effective.

Real-time calculation of ROP from controlled drilling parameters is highly effective in optimizing and giving advice on the drilling process. ROP data quality is very important for determining the optimum WOB and drillstring rotation to achieve minimum drilling costs [7]. Torque, WOB, drillstring rotation, drilling fluid circulation rate, and SPP are among the factors which highly affect the ROP during the drilling operation [8]. Due to the complexity of the drilling process, there is no reliable model to predict ROP from these surface measurements. There is a need for a model that can capture every factor contribution to predict ROP.

Several methods to predict ROP have been published either using empirical correlations or different artificial intelligence (AI) techniques. Maurer [9] developed an ROP empirical equation in which five inputs (drillstring rotation, WOB, bit diameter, rock strength, and drillability constant) were used to predict ROP for tri-cone bits for perfect cleaning conditions. Applying the Maurer model requires regression to define the formation drillability. Later on, the bit weight exponent was added to the model [10]. The Bingham model has only four input parameters, which made the model more accurate because of the utilization of the bit weight exponent. One of the most common models of predicting ROP was developed by Bourgoyne and Young [11] with nine inputs (depth, equivalent mud density, equivalent circulation density, WOB, drill bit size, drillstring rotation, Q, mud density, plastic viscosity) to account for many parameters, such as bit hydraulics and overbalance pressure. Multiple regressions are required to calculate seven different exponents for Bourgoyne and Young model. AL-AbdulJabbar et al. [12] developed a new ROP model with full consideration of different drilling parameters and mud rheological properties. They used nine inputs, and two exponents were calculated to reflect the bit exponent and the formation compressive strength. This approach helped to introduce the effect of the formation type and mechanical properties into the ROP model with different formations having a different compressive strength coefficient.

### Artificial Neural Network and its Application in Drilling Operation

An artificial neural network (ANN) is defined as an emulation of the biological neural system [13]. ANN has been used to solve computational challenges that linear computing techniques fail to handle [14,15]. The ANN structure is represented by the number of layers, and each layer has a basic element which is called a neuron. Neurons act as the fundamental processing elements of an ANN system. The minimum number for the ANN construction is three layers, which are the input, hidden, and output layer. Transfer functions are fundamentally used to link the ANN layers, and appropriate algorithms are used for data training [16]. In addition to that, constants known as model weights link the neurons in each layer with the subsequent layer neurons [17]. An optimization for the number of neurons is required because increasing the neuron number might cause overfitting and adversely affect the prediction performance. However, reducing the number of neurons may result in underfitting [18]. Usually, the input parameter values of an ANN model are normalized in the range of –1 to 1 [19]. During the training phase, backpropagation of errors and data processing are applied from the input layer all the way to the output layer. After that, the estimated output parameters are compared with the real outputs. For an efficient ANN model, the weights and biases of each layer are updated to estimated outputs with minimum error [20,21,22].

The ANN technique provides actual benefits to model and manage big data generated on a daily basis in the petroleum industry. ANN has many applications in the petroleum industry as summarized by Al-Bulushi et al. [23]. ANN has been applied in different aspects of petroleum engineering such as production forecasting [24,25], PVT (Pressure, volume, temperature) parameter prediction [26], well integrity evaluation [27], drilling fluid properties [28,29,30], reservoir, rock mechanics [31,32,33,34,35], drilling optimization [36,37,38,39,40,41], and permeability determination from well logs [42].

Khoukhi and Alarfaj [43] compared extreme learning machines (ELM) and radial basis function networks (RBF) on Bourgoyne and Young’s model and showed that RBF gave the best results in terms of accuracy and processing time. Amer et al. [44] used backpropagation feed-forward ANN to predict ROP with an R of 0.88 using 24 inputs, 50 neurons, and 1 layer. With more than 12,000 data points coming from six different wells, they obtained an R value of 0.88. Elkatatny [45] used an ANN feed-forward network to predict ROP on three offshore wells. The model was trained on 3333 data points from two wells with an R of 0.99 and average absolute percentage error (AAPE) of 5%. Then, 2700 data points were used to validate the model and resulted in the prediction of ROP with an R value of 0.99 and an AAPE of 4%. The rock unconfined compressive strength was constant in each section, which limited the model application to cases where there is no variation in the drilled rock strength. Kamel et al. [46] developed a technique for an automation rotary steerable system. 

The objective of this study is to use ANN to develop a new real-time ROP model using field data including drilling parameters and changing formation strength from three onshore wells. After data filtering and screening, one well was used to train and test the ANN model, while the other two wells were used to validate the developed model. A new empirical ROP equation was also derived based on the optimized ANN model and compared with four common ROP models.The developed equation was simplified to present the simplest form that can be used in the rig site without the need for the ANN code or the MATLAB lisence. 

In this manuscript, Section 2 represents the data description and Section 3 represents the development of the ANN model, including training, testing, validation, and the comparison with the published ROP correlations, in addition to converting the black box of the ANN to a white box by the development of a new ROP empirical correlation based on the optimized ANN model. Finaly, Section 4 represents the conclusion. 

## 2. Data Description

The field data were collected from 16-inch intermediate holes in three different vertical wells drilled onshore in the same carbonate formation using the same conventional bottom hole assembly (BHA). Only the parameters that are available in real-time will allow the real time determination of ROP. The data were collected from a real-time sensor based on footage. It is worth mentioning that formation of unconfined compressive strength (UCS) is included in the work as a continuous log rather than the average value for each formation type. Offset well logs were used after performing depth calibration to generate the UCS curve. Additionally, having mud logging in this section helped to reflect actual formation tops if it was found different. Based on that, the drilling parameters collected are ROP in ft/h, Q in gpm, drillstring rotation speed in rpm, SPP in psi, T in Klbf-ft, WOB in Klbf, and UCS in psi. 

Usually, field drilling data include all sorts of operations done on the 16-inch hole section such as drilling, tripping, and running the casing string. The first step was to capture only the drilling data where new footage was made and clean it from any other operations. This step requires a human interface using data filtering and elimination. For example, if the footage was 1000 ft, then suddenly the depth log shows 930 ft, then it is a trip out of the hole. Finally, only drilling data were considered in the data set used to develop the ROP model. 

**Figure 1:**
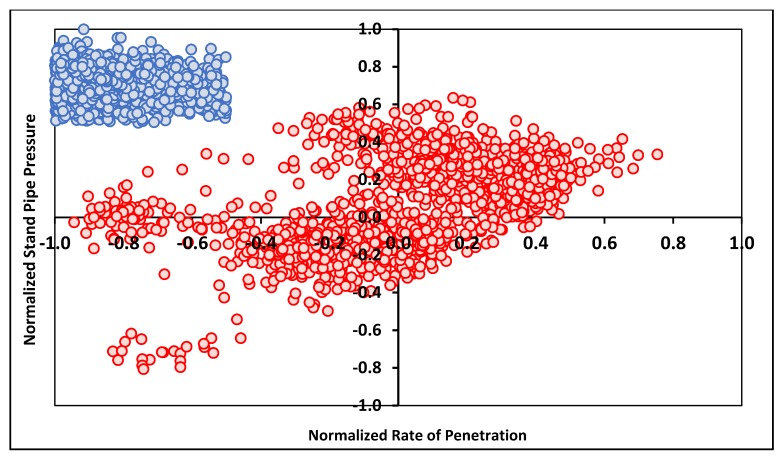
Example of data noise shown by clustering with the *x*-axis indicating normalized drilling rate of penetration (ROP) values in normalized stand pipe pressure.

The second step is to filter out any outliers by normalizing the data and checking the data cluster visually. Figure 1 shows one example of data clustering where the *x*-axis indicates the normalized ROP values and the *y*-axis indicates a normalized SPP. The values in blue color indicate very high SPP values at very low ROP, which may represent reaming operations or cement drilling. Based on that, the blue data cluster which represents a low ROP at a very high SPP was eliminated, as this is not normal in practical operation. Out of the 3311 data points in Well A, only 1528 data points (red data points in Figure 1) representing subsurface rocks penetration during drilling were used to train and test the ANN model. The red data points represent the common behavior of the ROP and SPP in normal drilling operation and indicate the progress of the drilling process.

**Figure 2:**
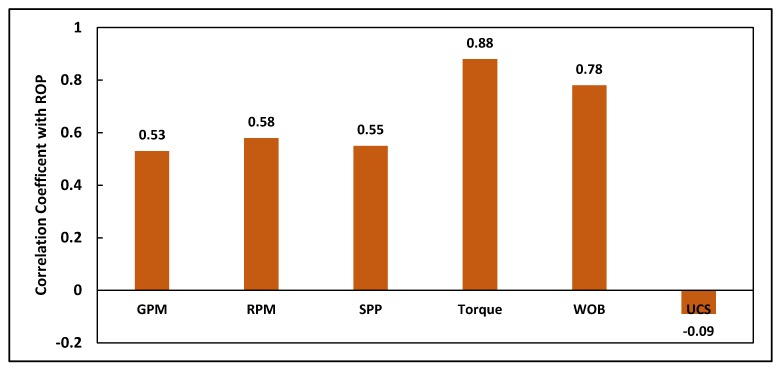
The correlation coefficient between the ROP and different drilling parameters collected from the 16” intermediate section in Well A.

For a better understanding of the cleaned data correlation, the correlation coefficients (R) of ROP with all other parameters from Well A are calculated and presented in Figure 2. The torque showed the maximum R value with ROP, 0.88, while the UCS has the lowest R value, −0.09. In addition, the ROP is a strong function of WOB where the R was 0.78, and it is a moderate function of Q, drillstring rotation, SPP where the R values were 0.53, 0.58, and 0.55, respectively. Although the R value of the UCS is very low, it is very important to include the UCS as an input parameter in building the ANN model, as it has a noticable effect on model accuracy [12].

The statistical parameters of the field data collected from the three wells are shown in Table 1, Table 2 and Table 3. For all parameters, the available data cover a wide range, which will help toward the development of an accurate prediction of ROP using ANN. Table 1 lists the data used for training the ANN model. The ROP ranges from 5 to 96.7 ft/h, which represents field practical values achieved usually in vertical sections. The flow rate ranges from 859.3 to 1110.6 gal/min, the drillstring rotation speed ranges from 76.9 to 157.9 RPM, the SPP ranges from 1396.3 to 2853.1 psi, the torgue ramges from 7.1 to 22 klbf-ft, the WOB ramges from 31.10 to 61.30 klbf, and the UCS ranges from 1821.1 to 42,819 psi, representing a different lithogy with different UCS values. 

Table 2 lists the statistical parameters for Well B which are used for validating the developed ANN model. ROP ranges from 5.4 to 153.7 ft/h, the flow rate ranges from 807 to 1124 gal/min, the drillstring rotation speed ranges from 51 to 119 RPM, the SPP ranges fom 1357 to 3827 psi, the torque ranges from 3.8 to 22.4 klbf-ft, the WOB ranges from 6.5 to 58 klbf, and the UCS ranges from 1996.2 to 28,190 psi. It is clear that some parameters have a wider data range than the one used for training, and this is a challenge to the developed model to assess its ability to predict the ROP with a data range out of the training data range. Table 3 lists the statistical parameters of Well C, which are used for validation of the developed model and also for model comparison with the published ROP models. ROP ranges from 16.79 to 126.66 ft/h, the flow rate ranges from 697.64 to 1171.40 gal/min, the drillstring rotation speed ranges from 64.52 to 168.86 RPM, the SPP ranges fom 676.17 to 2628.60 psi, the torque ranges from 12.9 to 23.29 klbf-ft, the WOB ranges from 26.17 to 58.33 klbf, and the UCS ranges from 5087.20 to 28,190 psi. The same behavior was noted as Well B, as the data range of some parameters is outside the data range of the training parameters of Well A.

Surprisingly for all wells, however, the UCS profile in the field data represents different rock strength within the same formation, and it has the lowest R value with ROP. For example, in Well A, the UCS ranges from 1821.1 to 42,819 psi, while its correlation coefficient with ROP is −0.09. The same behavior was noticed for Wells B and C.

## 3. ANN Model 

### 3.1. Model Development and Results 

**Figure 3:**
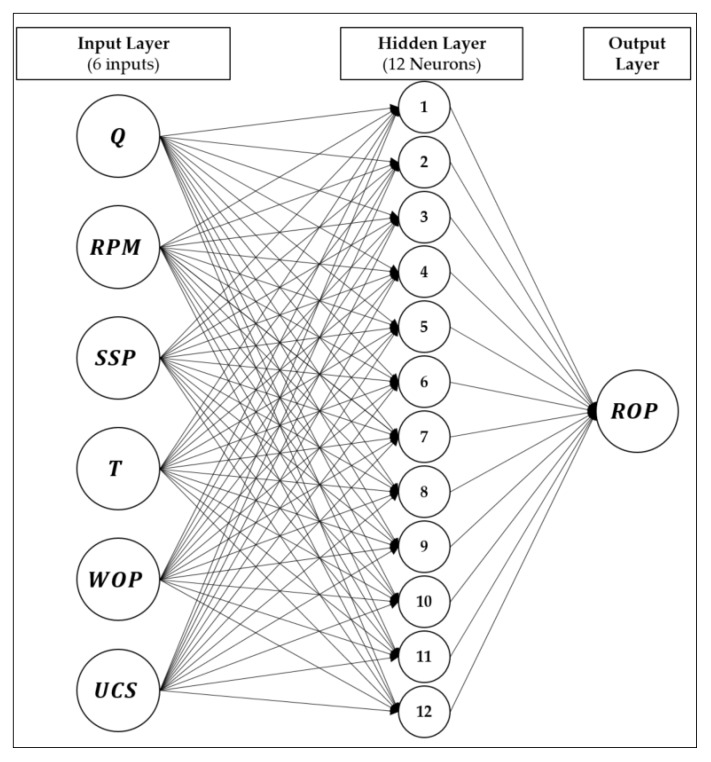
Artificial neural network (ANN) model structure for ROP prediction.

The ROP model was built using an ANN feed-forward network with the six input parameters discussed in the previous section. The ANN model consists of 12 neurons and one hidden layer, as shown in Figure 3. The optimum number of neurons and layers was selected based on achieving the minimum AAPE and maximum correlation coefficient as the two governing factors through several trials. During the trials, the minimum number of neurons was 6, and the maximum was 20. Only one hidden layer was used, as having two or more layers did not improve the results even with a different number of neurons. In addition, using one layer reduces the size of the ANN model correlation matrix of weights and biases. The Levenberg–Marquardt training function (trainlm) was used as a training function, while pure-linear was used as a transfer function. 

**Figure 4:**
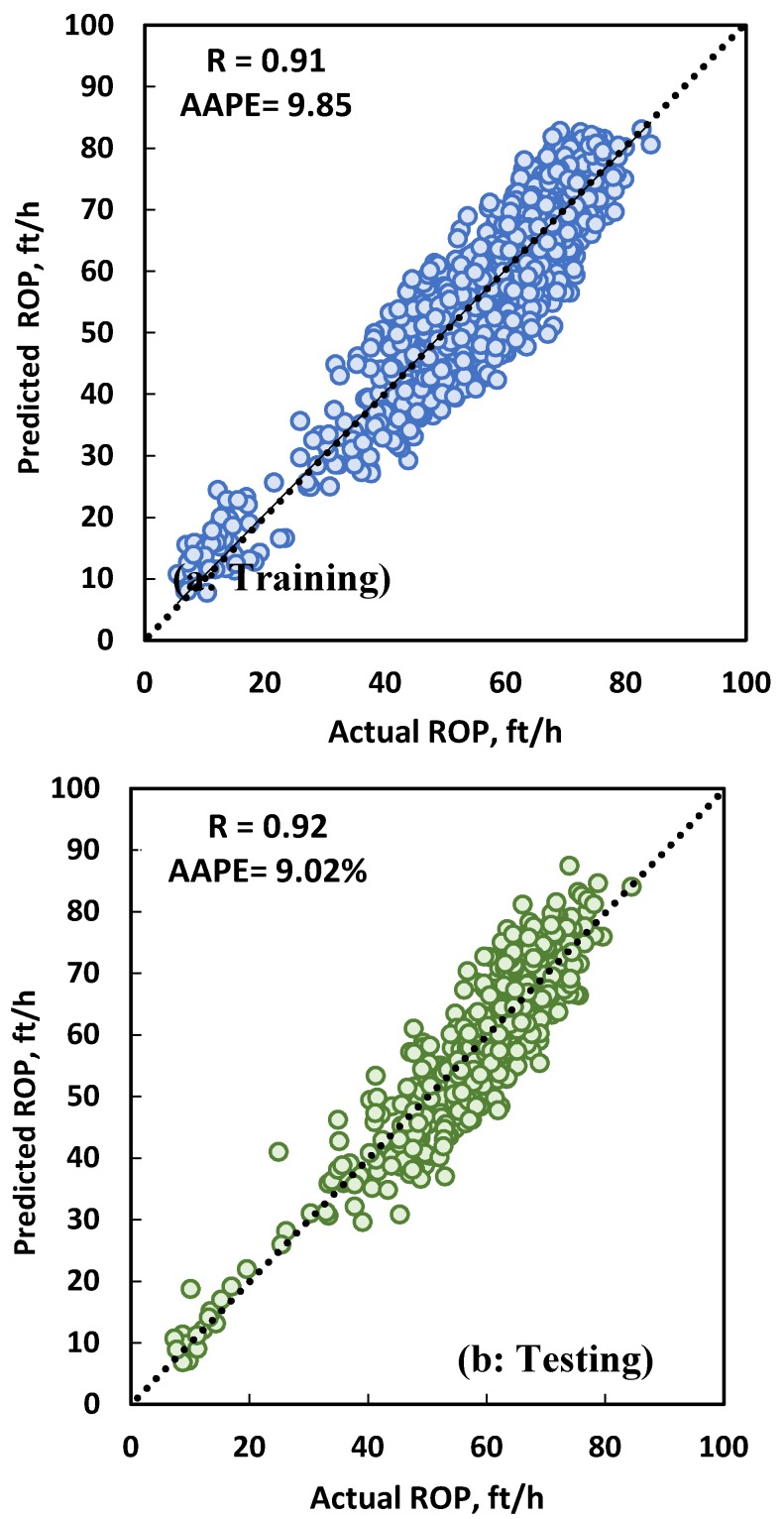
Actual ROP vs. predicted ROP from the ANN model using 1528 data points from Well A with 70% for training (**a**) and 30% for testing (**b**).

**Figure 5:**
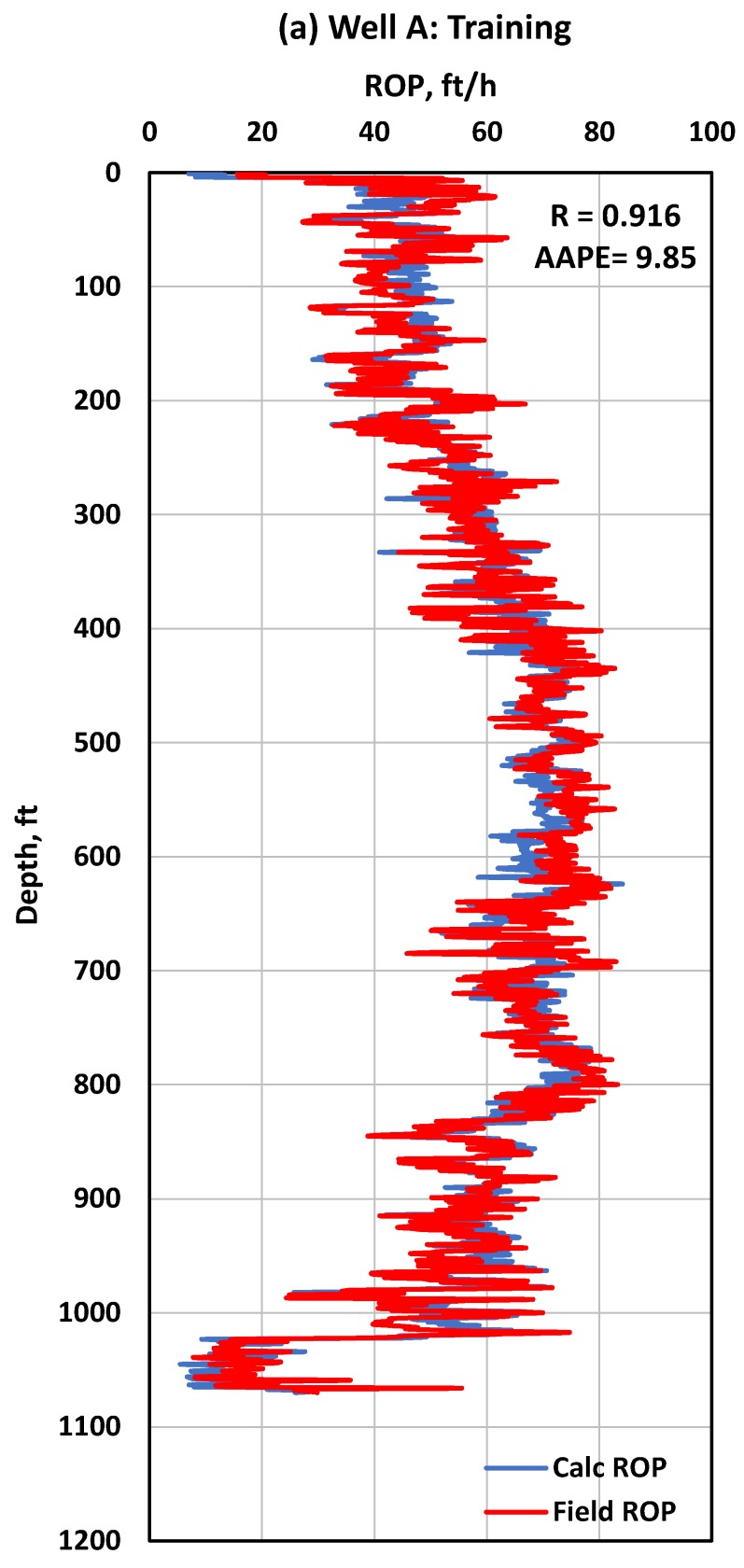

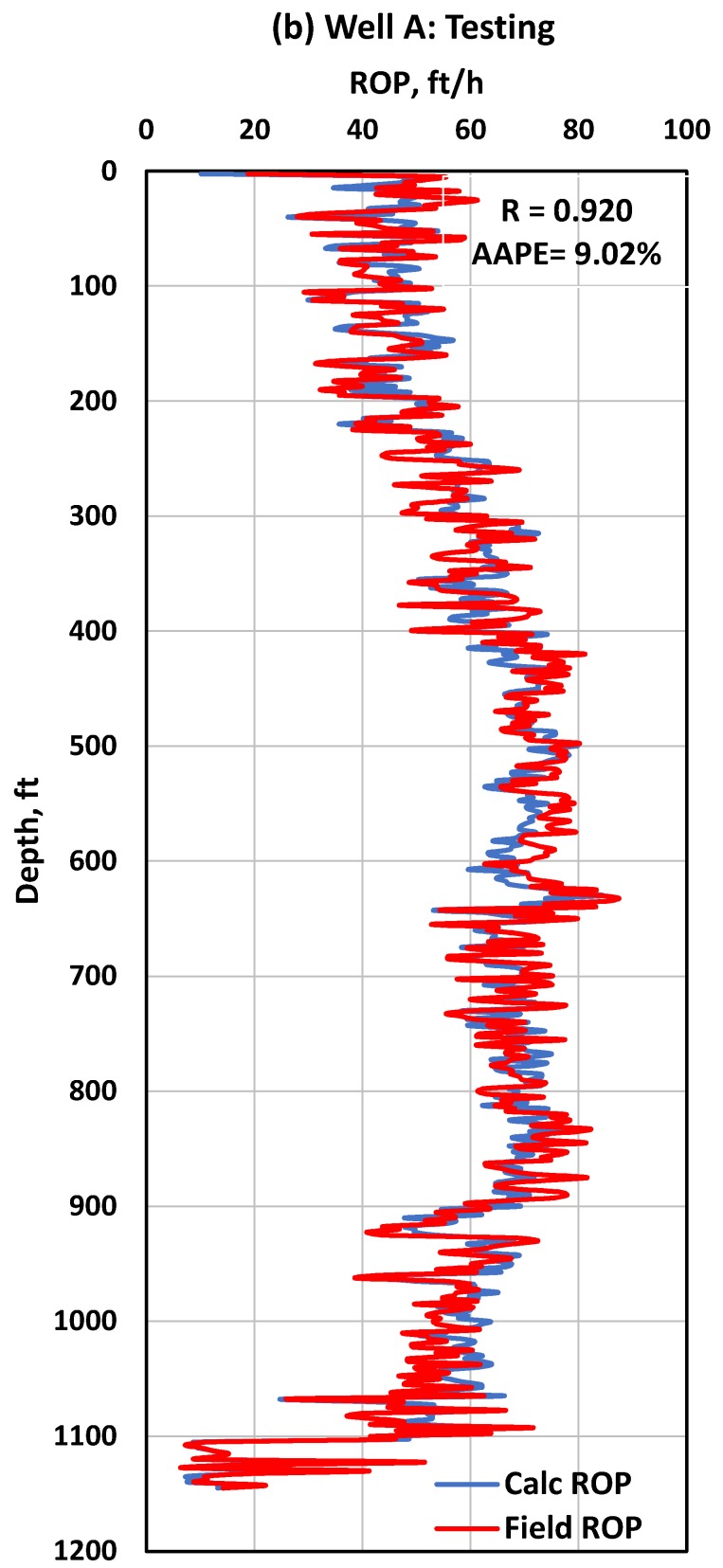
Complete profile of actual ROP vs. predicted ROP for (**a**): the ANN model training and (**b**) ANN model testing, using 1528 data points from Well A.

The data collected from Well A (1528 data points) were used to train the ANN model using one hidden layer, 12 neurons, the trainlm training function, and pure-linear transfer function. Data from Well A were selected for training because of the wider range of parameters available in the data set compared to Wells B and C which allow the ANN model to learn better about ROP trends in this field. A total of 70% of the data was used for training and 30% for testing the developed model. For the training dataset, an R value of 0.92 and an AAPE of 9.85% were achieved (Figure 4a), while for testing, an R value of 0.92 and an AAPE of 9.02% were achieved (Figure 4b). A complete ROP profile is shown in Figure 5 for both ANN model training and testing. It is clear from the visual check that the developed ANN model for ROP prediction has a high accuracy where the predicted and actual ROP lines are overlapped, and also the predicted line follows the trend of the actual ROP line when there is an increase or decrease. One of the main reasons for the change in ROP values is the change in UCS values, and the developed model was able to capture that affect as shown in Figure 5 for both training and testing. 

**Figure 6:**
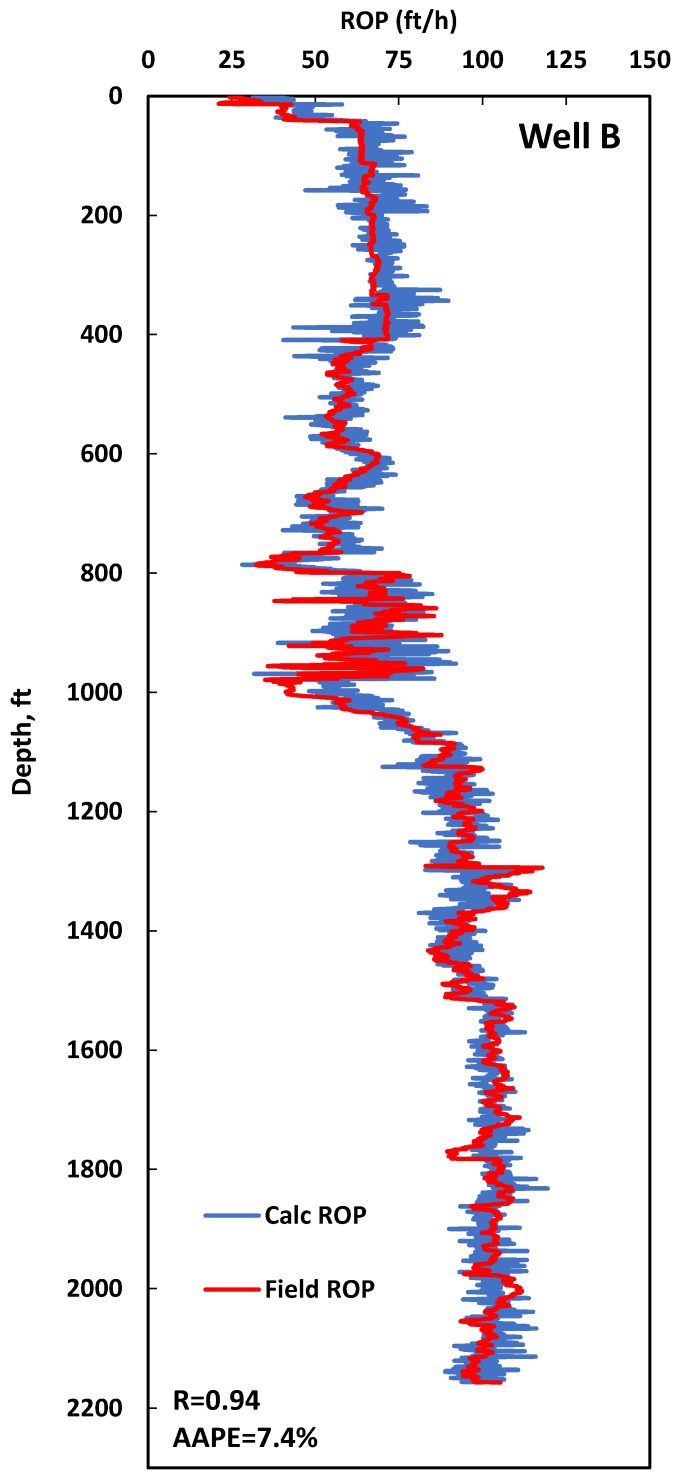

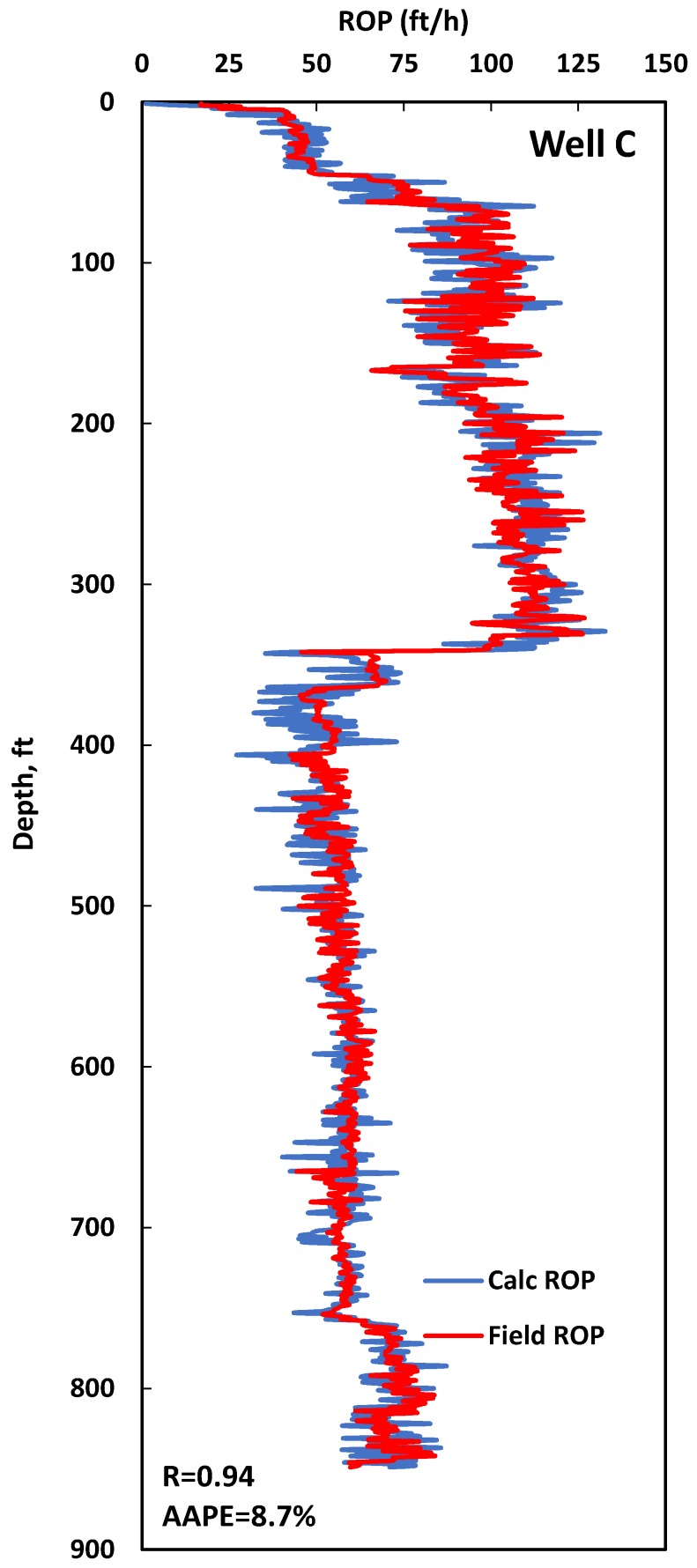
Complete profile of actual ROP vs. predicted ROP from the developed ANN model using data from Wells B and C. Well-B data was used for evaluation the developed ROP model, while Well-C data was used for validating and comparison with other published ROP models.

The datasets collected from Wells B and C, 2157 and 849 data points, respectively, were used to validate the developed ANN model for ROP prediction. The ROP model yielded an R of 0.95 and 0.94 between the actual and predicted ROP and AAPEs of 7.4% and 8.7% for Wells B and C, respectively. The calculated ROP was compared with the actual ROP for both Wells B and C, as shown in Figure 6. It is clear in Figure 6, which represents Well C data, that there is a big change in the ROP values, which resulted from the change of the UCS, as this section consists of different carbonate formations where each formation has its own UCS value. This is the main reason the UCS was included as an input, and it was proven that it has a big effect. The developed ANN–ROP model was able to capture the big variation in the UCS and yielded a strong ROP model that can capture the increase and the decrease of the actual ROP value over a wide depth range. Even though Well C has a greater data range than that of the training data, the model was able to predict the ROP with a high accuracy. Because of the big variation in the ROP of Well C, this well was used for comparing the developed ANN model with the previous ROP correlations.

### 3.2. ANN Model Empirical Correlation

To permit real-time prediction, the current black box model has to be converted to a white box model to enable ROP prediction outside the AI modeling software. An empirical correlation can be derived from the ANN model to represent the ANN structure based on the optimized ANN model. These types of correlations are based on sets of weights and biases that can be written as a matrix. The weights and biases can be transferred to an empirical equation through a mathematical operation to enable easier utilization of the developed model by engineers. In the ANN model, each neuron handles the following functions [47]:

i- Multiplication of the input parameters, x_1_, x_2_, x_3_, ... x_n_ by the associated input weights;
ii- Summation of the weight and input product to the bias value associated with the neuron;
iii- The passage of the summation result, u, through an activation function (linear or nonlinear transformation), Φ. The transfer function can be logistic sigmoid (logsig), hyperbolic tangent sigmoidm (tansig), or linear (purelin), as described in Table 4.

The neuron’s output (y) is the result of the action of the activation function, as follows:

(1) $$y=\Phi \left(\overset{n}{\underset{i=1}{\sum }}x_{i}w_{i}+b\right)=\Phi \left(w^{T}x+b\right)$$

For a one-layer ANN structure (plus one hidden), the equation can be written as:

(2) $$y=\Phi_{2}\left(\overset{m}{\underset{j=1}{\sum }}w_{j1}\Phi_{1}\left(\overset{n}{\underset{i=1}{\sum }}w_{ij}x_{i}+b_{j}\right)+b_{1}\right)\;$$

where *m* is the number of neurons and *n* is the number of inputs. If the linear transfer function was used on both the first layer and hidden layer, the above equation can be written as:

(3) $$y=\left(\overset{m}{\underset{j=1}{\sum }}w_{j1}\left(\overset{n}{\underset{i=1}{\sum }}w_{ij}x_{i}+b_{j}\right)+b_{1}\right)$$

In MATLAB language, Equation (3) can be written as:

(4) $$y=LW\left(IW\times X+b_{1}\right)+b_{2}=\left(LW\times IW\right)\times X+\left(LW\times b_{1}\right)+b_{2}\;$$

where:

*m*: Number of neurons
*n*: Number of inputs
*X*: The input matrix (*x*_1_, *x*_2_, …, *x*_n_)
*LW*: Layer weights matrix, [1, m]
*IW*: Input weights matrix, [m, n]
*b*_1_: First layer bias matrix, [m,1]
*b*_2_: Second layer bias, scalar

There will be only one coefficient (*a*) for each input (x). Thus, the coefficient matrix of the ANN model can be written as:

(5) $$\left[\begin{array}{ccc} a_{1} & a_{2} & a_{3} \end{array}\begin{array}{cc} \ldots & a_{n} \end{array}\right]=\left(LW\times IW\right)$$

while a constant (*c*) can be represented by:

(6) $$c=\left(LW\times b_{1}\right)+b_{2}$$

Then, the final empirical correlation model will be:

(7) $$y=a_{1}x_{1}+a_{2}x_{2}+a_{3}x_{3}+\ldots +a_{n}x_{n}+c\;$$

Substituting the *x* values with corresponding *ROP* model normalized input parameters results in the following *ROP* empirical correlation:

(8) $$ROP_{n}=a_{1}Q_{n}+a_{2}RPM_{n}+a_{3}SPP_{n}+a_{4}T_{n}+a_{5}WOB_{n}+a_{6}UCS_{n}+c\;$$

where the values of *a*_1_–*a*_6_ are listed in Table 5, and the normalized parameters can be defined from the following equations:

(9) $$ROP_{n}=0.0248ROP-1.1712$$

(10) $$\;Q_{n}\;=0.008Q-7.8388\;$$

(11) $$RPM_{n}\;=0.0248RPM-2.9084$$

(12) $$\;SSP_{n}=0.0014SSP-2.9169$$

(13) $$\;T_{n}\;=0.1408T-2.0986\;$$

(14) $$WOB_{n}=0.0671WOB-3.1141\;$$

(15) $$UCS_{n}\;=0.0001UCS-1.1516\;$$

Combining Equations (8)–(15) with constants in Table 5 gives the final *ROP* equation (Equation (16)), which can be used directly to calculate *ROP* from input parameters.

(16) ROP=0.01463 Q+0.28979 RPM+0.00522 SSP+4.1812 T+0.4782 WOB+0.000398UCS−104.957

### 3.3. Model Comparison

To compare the obtained results from the ANN model with the previous ROP models, four ROP models were selected, and a comparison was performed between all of them using Well C data (849 data points). For the Maurer model [9] using Well C data, the formation drillability constant (K) was found using regression to be 6.7275 × 10^−7^, while for the Bingham [10] model, three constants K, a_WOB_, and b_WOB_ were calculated using regression to be 0.2, 1.589, and 0.89, respectively. The third model is Bourgoyne and Young [11], for which the seven exponents, *a*_1_–*a*_7_, were found to be 2.3583, 2 × 10^−4^, −1.0 × 10^−4^, 1.06 × 10^−7^, 0.636, 0.6259, and −0.1109, respectively. The final ROP model is the Al-AbdulJabbar et al. [12] model, where only two constants, *a_(WOB)_* of 0.854 and *b_(UCS)_*, ranged between 1.189 and 1.275 depending on formation type. 

**Figure 7:**
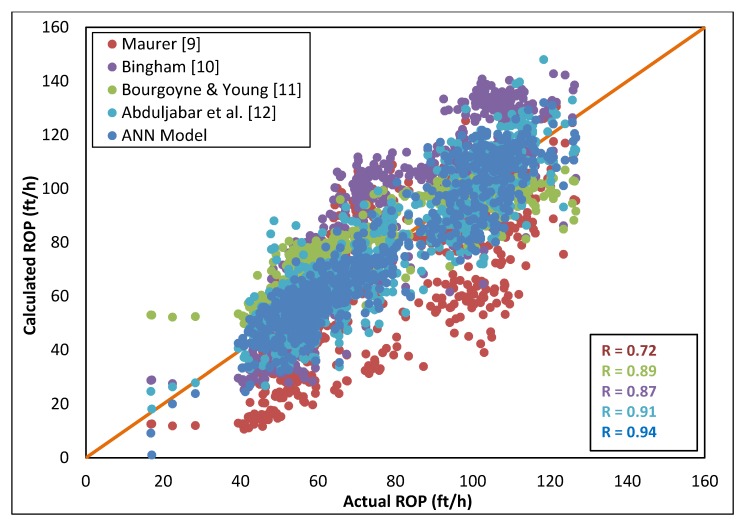
Cross-plot of actual vs. calculated ROP using different ROP correlations using the data set from Well C.

Figure 7 shows that the developed correlation outperformed all other models with the highest R value (0.94) as compared with the Maurer model [9] which yielded an R value of 0.72, the Bingham [10] model wich yielded an R value of 0.87, Bourgoyne and Young [11] which yielded an R value of 0.89, and Al-AbdulJabbar et al. [12] which yielded a high R value of 91 as it considered the effect of clustering based on the UCS value.

**Figure 8:**
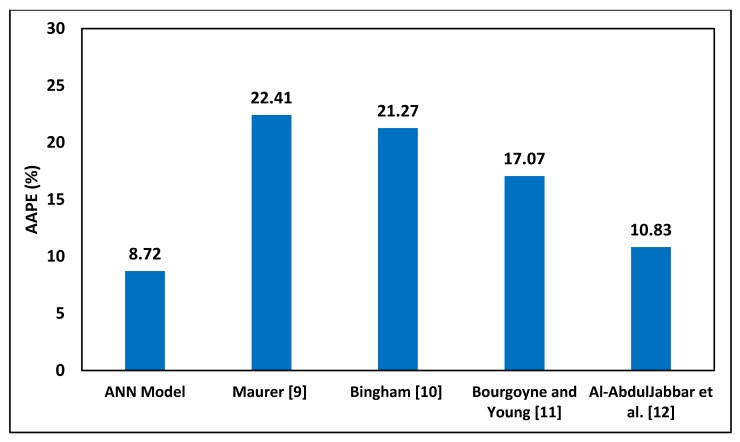
AAPE for different ROP correlations applied to Well C data for comparison with the developed correlation.

In terms of the average absolute percentage error, Figure 8 shows that the developed empirical correlation of ROP yielded the lowest AAPE, which was 8.72%, while the Maurer model [9] yielded an AAPE of 22.41%, the Bingham [10] model yielded an error of 21.27%, Bourgoyne and Young [11] yielded an AAPE of 17.07%, and Al-AbdulJabbar et al. [12] yielded a high AAPE of 10.83%. Based on these results, the developed ROP equation based on the optimized ANN model can be used as a robust and precise equation to predict ROP in future wells in the same field while the developed equation can be tuned for wells in other fields. 

## 4. Conclusions

Field data collected during drilling operation of three vertical onshore wells drilled in the same carbonate formation using the same conventional bottom hole assembly were used to train, test, and validate an ANN model to predict ROP. ROP was successfully predicted as a function of T, Q, drillstring rotation, WOB, SPP, and UCS with an AAPE of 9.85% and 9.02% for training and testing, respectively, and the correlation coefficient was above 0.91 for training and testing. The model was able to calculate ROP for the selected sections in Wells B and C drilled in the same formation with an R value of 0.94 for the two wells and an AAPE of 7.4% and 8.7%, respectively. A simple and highly accurate ROP empirical equation was developed based on the weights and biases of the optimized ANN model so that field engineers can apply it in daily calculations very easily. In addition, the developed ROP equation was compared with four existing ROP correlations using data from Well C, which was not used during the model development. The new correlation outperformed all other correlations with the lowest AAPE and highest R value, which can allow field engineers to use it to predict accurate ROP in future wells or define a certain parameter to achieve a desired ROP.

The main limitation of the developed model is the data range of the used training data where the model can be uded to predict the ROP with a high accuracy for any new sets of data that have the same data range as that of Well A. Moreover, it is recommended to apply this model for the vertical section only, where carbonate formation exists. For other well profiles, there is a need to develop another ROP.

## Figures and Tables

**Table 1:** Statistical parameters of the drilling parameters collected from the 16” intermediate section of Well A.

Statistical Parameter	ROP (ft/h)	Q (gal/min)	Drill String Rotation Speed (RPM)	SPP (psi)	Torque (Klbf-ft)	WOB (Klbf)	UCS (psi)
**Minimum**	5.00	859.30	76.9	1396.3	7.1	31.10	1821.1
**Maximum**	96.70	1110.60	157.9	2853.1	22	61.30	42,819
**Mean**	57.68	998.95	140.02	2291.2	16.18	48.78	13,354
**Kurtosis**	−0.82	−1.30	−1.31	−0.50	−1.06	−0.73	1.79
**Skewness**	3.42	4.02	5.84	3.28	4.34	3.54	7.34

**Table 2:** Statistical parameters of the drilling parameters collected from the 16” intermediate section of Well B.

Statistical Parameter	ROP (ft/h)	Q (gal/min)	Drillstring Rotation Speed (RPM)	SPP (psi)	Torque, (Klbf-ft)	WOB (Klbf)	UCS (psi)
**Minimum**	5.4	807	51	1357	3.8	6.5	1996.2
**Maximum**	153.7	1124	119	3827	22.4	58	28,190
**Mean**	106.87	1028.6	101.86	3023	15.13	40.15	11,654
**Kurtosis**	−0.31	−1.51	−2.57	−0.6695	−0.31	−0.03	0.97
**Skewness**	1.97	3.56	12.04	2.51	2.31	2.33	5.52

**Table 3:** Statistical parameters of the drilling parameters collected from the 16” intermediate section of Well C.

Statistical Parameter	ROP (ft/h)	Q, (gal/min)	Drillstring Rotation Speed (RPM)	SPP (psi)	Torque (Klbf-ft)	WOB (Klbf)	UCS (psi)
**Minimum**	16.79	697.64	64.52	676.17	12.90	26.17	5087.20
**Maximum**	126.66	1171.40	168.86	2628.60	23.29	58.33	28,190
**Mean**	72.98	890.46	129.22	1664.40	18.25	45.19	11,382
**Kurtosis**	0.59	0.33	−0.20	0.44	0.80	−0.51	1.30
**Skewness**	2.07	1.26	2.97	1.95	3.20	2.55	7.77

**Table 4:** Different transfer function definition.

Transfer Functions	Definition
logsig	$\Phi =\frac{1}{1+e^{-u}}$
tansig	$\Phi =\frac{2}{1+e^{-2u}}-1$
purelin	Φ=u

**Table 5:** Constants of the *ROP* empirical correlation (Equation (8)) for ANN models’ different transfer function definition.

$a_{1}$	$a_{2}$	$a_{3}$	$a_{4}$	$a_{5}$	$a_{6}$	c
0.04535	0.28979	0.09246	0.17677	0.73647	0.09868	−0.06879

## List of Symbols

T	Torque, klbf-ft
Q	Flow rate, gpm
R	Correlation coefficient
h	Hour
m	Number of neurons
n	Number of inputs
X	The input matrix
LW	Layer weights matrix
IW	Input weights matrix
b	Bias matrix
a	Coefficient
c	Constant
Φ	Activation function

## List of Abbreviations

ROP	Rate of penetration, ft/h
UCS	Uniaxial compressive strength, psi
WOB	Weight on bit, klbf
RPM	Revolution per minute
AAPE	Average absolute percentage error
SPP	Stand pipe pressure, psi
ANN	Artificial neural network
